# Biological Activity of Extracts from Aromatic Plants as Control Agents against Spoilage Molds Isolated from Sheep Cheese

**DOI:** 10.3390/foods10071576

**Published:** 2021-07-07

**Authors:** Nuria Muñoz-Tebar, Emilio J. González-Navarro, Teresa María López-Díaz, Jesús A. Santos, Gonzalo Ortiz de Elguea-Culebras, M. Mercedes García-Martínez, Ana Molina, Manuel Carmona, María Isabel Berruga

**Affiliations:** 1 Food Quality Research Group, Institute for Regional Development (IDR), Universidad de Castilla-La Mancha, 02071 Albacete, Spain; nuria.munoz@uclm.es (N.M.-T.); emiliojose.gonzalez@uclm.es (E.J.G.-N.); ana.molina@uclm.es (A.M.); manuel.carmona@uclm.es (M.C.); 2 Department of Food Hygiene and Food Technology, Veterinary Faculty, Universidad de León, Campus de Vegazana s/n, 24071 León, Spain; teresa.lopez@unileon.es (T.M.L.-D.); j.santos@unileon.es (J.A.S.); 3 Centro de Investigación Agroforestal de Albaladejito (IRIAF-JCCM), Carretera Toledo-Cuenca km 174, 16194 Cuenca, Spain; gonzaloo@jccm.es; 4 Catedra de Química Agrícola, Escuela Técnica Superior de Ingenieros Agrónomos y de Montes, Universidad de Castilla-La Mancha, Campus Universitario s/n, 02071 Albacete, Spain; mariamercedes.garcia@uclm.es

**Keywords:** *Penicillium*, *Aspergillus*, molecular identification, essential oils, ethanolic extracts from solid by-products, antifungal activity, antioxidant activity, mycotoxins

## Abstract

The aim of this work was to assess the antifungal and antioxidant activity of essential oils and ethanolic extracts from distilled solid by-products from aromatic plants (*Artemisia* *dracunculus*, *Hyssopus* *officinalis*, *Lavandula* *stoechas*, *Origanum* *vulgare* and *Satureja* *montana*) against 14 fungi strains isolated from sheep cheese and identified at species level using DNA barcoding based on β-tubulin sequence analysis. In addition, capacity of fungi to produce ochratoxin A, patulin, cyclopiazonic acid and sterigmatocystin was analyzed. Of the isolates, 85.7% belonged to *Penicillium* (*P*. *commune*/*biforme*, *P*. *crustosum*) and 14.3% to *Aspergillus* (*A*. *puulaauensis* and *A*. *jensenii*), the first time that these *Aspergillus* species have been found in sheep’s cheese. All *P*. *commune* isolates were producers of cyclopiazonic acid, and the two *Aspergillus* strains produced sterigmatocystin, but the others did not produce any tested mycotoxin. Among the essential oils tested, oregano, savory and tarragon had a significant antifungal activity against all the isolated strains, but no ethanolic extract showed antifungal activity. By contrast, ethanolic extracts showed great potential as antioxidants. The identification of new molds in cheese will help the dairy industry to know more about those molds affecting the sector, and the use of aromatic plants in the control of fungal spoilage could be a suitable alternative to chemical preservatives used in the agri-food industry.

## 1. Introduction

Molds are one of the main microorganisms that cause food spoilage and hence their control is one of the major concerns for the food industry since they are responsible for significant economic losses [1,2]. In addition, the mycotoxins produced by some types of molds are potentially toxic and can cause serious problems related to food safety [3,4].

In this sense, cheese can be considered as a good substrate for many fungi species and its ripening and storage make this dairy product even more susceptible to growth and fungal contamination. Spoilage by filamentous molds in cheese causes visible alterations that can lead to the formation of off-flavors harming the quality, as well as affecting the sensory characteristics or producing toxic secondary metabolites knowns as mycotoxins [5,6]. Among the spoilage and contaminating molds, the main mycotoxin-producing isolates in cheese are those belonging to the genus *Penicillium* and, to a lesser extent, *Aspergillus* [7,8,9,10,11]. There are also other less common molds such as *Alternaria*, *Fusarium*, *Geotrichum* and *Mucor* that can cause several surface defects in cheese [12]. For this reason, the identification of the mycobiota that affects cheeses plays a key role in food safety and can help the dairy industry improve control measures against these fungi. 

Conventional identification of fungi has been carried out by phenotypic criteria that includes microscopic and morphological analysis, difficult to interpret by non-experts. Besides, the procedure normally takes several days and depends on optimal growth and sporulation in a specific culture media that can result in confusing identification of closely related species [13]. Therefore, molecular identification methods based on polymerase chain reaction (PCR) have emerged in recent years. One of the main reasons for this is that they offer the advantage of measuring genotypic characteristics and do not depend on culture conditions [14]. Among these, sequence analysis can be highlighted, which allows a global approach to fungal identification [13]. In this context, DNA barcoding is a sequence analysis technique with great accuracy that allows the identification (species level) of a wide range of microorganisms, providing an effective and rapid acquisition of molecular data [15] that can be compared with a reliable DNA sequences database [16] in order to identify new fungi within the food industry in a faster and more accurate manner.

The increasing demand for more natural and chemical-free foods by consumers, as well as their growing concern about the environment, is changing the agri-food industry. Currently, some consumers consider chemical food additives as a potentially unhealthy risk to human health [17], thus the need to find natural compounds that can replace synthetic ones has arisen, and researchers have focused on finding sources of compounds with biological activities that are safer and more environmentally friendly. In relation to this, essential oils are a good alternative since they possess antioxidant properties [18,19] as well as antifungal and antibacterial activity [20,21,22,23,24], in addition to being a natural source and being generally recognized as safe (GRAS) by the US Food and Drug Administration (FDA). Essential oils are products obtained from whole plants, flowers, seeds or peels by steam distillation or hydro-distillation, highly soluble in organic solvents but insoluble in water and usually colorless [25]. Once essential oil has been extracted from aromatic plants, a large amount of solid residue is generated, a major environmental concern if not properly controlled. However, these solid by-products from the distillation of essential oils are a source of bioactive compounds such as polyphenols [26,27]. These compounds can be used in the food industry for the development of new functional foods and their use can increase the profitability of aromatic plants and improve resource efficiency by promoting the circular economy.

The aims of the present work are: (i) to isolate contaminating molds from the surface of sheep cheeses in order to identify them, characterize the fungi that affect this type of cheese and contribute to the knowledge of these contaminants, and (ii) to evaluate the in vitro antifungal and antioxidant activity and the feasibility of using essential oils and ethanolic extracts from distilled solid residues in the dairy industry to control the fungal growth that causes spoilage and important economic losses in this sector.

## 2. Materials and Methods

### 2.1. Cheese Samples and Mold Isolation

Twenty-one visually different samples of filamentous molds from six farms, one of them with two maturation locations (1A and 1B), located in the Castilla-La Mancha region (Spain) were randomly obtained from two-months-ripened semi-hard pressed sheep cheese surfaces for isolation in the laboratory. The cheese making process was similar on all six farms, as it is a typical regional process. The starter culture used was commercial and composed of *Streptococcus lactis*, *Streptococcus cremoris* and *Streptococcus thermophilus* and the concentration of the brine used was 18% *w*/*v* NaCl. All the farms used similar ripening conditions (temperature 10–12 °C/80% RH).

The mold samples were cultured on Potato Dextrose Agar, PDA (Merck, Darmstadt, Germany) plates at 25 ± 1 °C for 7 days [2] and, after incubation, up to three colonies per sample were selected and inoculated again on PDA plates until pure cultures were obtained. Finally, the 14 molds that grew correctly under laboratory conditions and were successfully isolated were preserved at 4 °C on Malt Extract Agar, MEA (VWR Chemicals, Radnor, PA, USA) slants until molecular identification or antifungal assays.

### 2.2. Molecular Identification of Molds by DNA Barcoding

For molecular identification, the pure molds were cultured on MEA slants at 25 ± 1 °C for 7 days according to Ramos-Pereira et al. [11] and the identification at genus and species level of the isolates was carried out using DNA barcoding analysis. For this, the mycelium was collected with 5 mL of sterile Tween 80 (0.05% *v*/*v*) and 2 mL were transferred to an Eppendorf and centrifuged at 16,000× *g* for 3 min. The pellet obtained was washed twice with 1 mL of Milli-Q water and suspended in 250 μL of Instagene matrix (Bio-Rad, CA, USA). Then, the DNA was extracted for 3 h by heating the samples at 56 °C followed by 10 min at 95 °C. Subsequently, the sample was vortex mixed and centrifuged at 12,000× *g* for 3 min, the supernatant was transferred to a new Eppendorf tube and 5 μL were taken for PCR amplification [13]. Polymerase Chain Reaction (PCR) was carried out in 25 μL reactions in a Mastercycler Personal (Eppendorf Iberica, Madrid, Spain) and the amplification of β-tubulin gene was carried out using primers Bt2a 5′-GGT AAC CAA ATC GGT GCT GCT TTC-3′ and Bt2b 5′-ACC CTC AGT GTA GTG ACC CTT GGC-3′ with the following profile: 94 °C/5 min and 35 cycles of 94 °C/45 s, 55 °C/45 s and 72 °C/60 s, and a final elongation at 72 °C/7 min, as described by Visagie et al. [16]. PCR’s products were purified using NucleoSpin Gel and PCR Clean-up Kit (Macherey-Nagel, Düren, Germany) and both strands were sequenced in a Mega-BACE 500 sequencer (GE Healthcare Life Sciences, Hatfield, UK). The sequences obtained were visually checked using Chromas Lite 2.1 software (http://technelysium.com.au/; accessed on October 2019) and strain identification was carried out by BLAST search against a verified database for β-tubulin [16].

Phylogenetic tree construction was performed with the MEGA X software (https://www.megasoftware.net/; accessed on October 2019) by alignment with ClustalW and using UPGMA clustering and distance estimation with the 2-parameter Kimura model, and Bootstrap analysis with 1000 replicates. In addition to the sequences of the analyzed strains, verified sequences of the identified species and others regularly present in cheeses were included.

### 2.3. Analysis of Mycotoxins Production

Yeast extract, sucrose, MgSO_4_·7H_2_O, CuSO_4_·5H_2_O, ZnSO_4_·7H_2_O and agar for making Yeast Extract Sucrose agar (YES, [28]) were acquired from VWR (VWR Chemicals, Radnor, PA, USA). Cyclopiazonic acid (CPA), patulin (PAT) and sterigmatocystin (STC) were purchased from Sigma (Sigma-Aldrich), ochratoxin A (OTA) from Cayman Chemical (Cayman Chemical Company, Ann Arbor, MI, USA). 

The mold strains were cultured on YES plates [28] for 7–14 days for the analysis of the production of the three more common mycotoxins (OTA, CPA, PAT) that can be found in cheese [7], and sterigmatocystin (STC) which is a mycotoxin produced by *Aspergillus* of the *Versicolores* section [29]. The detection of mycotoxin production was carried out by Thin Layer Chromatography (TLC) using the agar plug technique described by Samson et al. [30]. For OTA and PAT analysis, aluminum plates (silica gel 60 F254, Merck, Darmstadt, Germany) were directly used, but for CPA detection the plates were previously submerged into oxalic acid in methanol (10%) for 2 min and heated in an oven at 110 °C for a further two min [31]. Then, the plates were inoculated with the molds by using a Camag Nanomat 4 (Camag, Muttenz, Switzerland). The mobile phase of TLC analysis was TEF (toluene/ethyl acetate/90% formic acid, 5:4:1) [30]. Finally, the plates were dried and treated as follows regarding the type of mycotoxin: OTA (NH_3_ vapors for 2 min; fluorescent blue-turquoise spots were observed under ultraviolet light) [32]; CPA (pulverization with Ehrlich reagent; a violet-blue spot was observed after some minutes) [31]; PAT (pulverization with 0.5% *v*/*v* 3-methyl-2-benzothiazolinone hydrazone (MBTH) and heating in an oven at 105 °C during 10 min; a yellow spot appears in visible light [32]; STC (pulverization with 20% *w*/*v* AlCl_3_.6H_2_0 in ethanol and heating at 80 °C for 10 min, a bright yellow spot appears under UV light) [33]. The detection limit of the mycotoxins tested was 10 μg/mL for CPA, PAT and OTA, and 50 μg/mL for STC.

### 2.4. Obtainment of Essential Oils and Ethanolic Extracts from Aromatic Plants

Oregano (*Origanum vulgare*), hyssop (*Hyssopus officinalis*), savory (*Satureja montana*) and tarragon (*Artemisia dracunculus*) plants were purchased from Josenea Bio (Navarra, Spain) and Spanish lavender (*Lavandula stoechas*) from Mama Juana (Murcia, Spain).

Leaves were grounded, passed through a sieve with 0.5–1 mm mesh and 100 g of dry matter from each plant was humidified for 24 h until saturation, before distillation. Subsequently, they were introduced into the reactor and steam distilled for three h. Once the process was completed, the essential oils (EOs) on top of the aqueous layer were separated and dried with anhydrous sodium sulfate and stored at 4 °C in amber glass vials until chemical characterization and assessment. The resulting residual plant material was dried in an oven with forced ventilation (Heraeus, Hanau, Germany) at 35 °C for 24 h for further processing as ethanolic extracts (EEs).

EEs of the solid by-products resulting from the production of the essential oils of oregano, hyssop, Spanish lavender, savory and tarragon were obtained in a Soxhlet apparatus. One hundred grams of the solid residue from the essential oil extraction was weighed on a filter paper cartridge and introduced into a Soxhlet extractor. The extraction was carried out with 400 mL of 60% *v*/*v* ethanol for five h and ethanolic extracts were recovered and allowed to cool to room temperature. The EEs were vacuum filtered with filter paper, and centrifuged in an Eppendorf centrifuge 5804R (Eppendorf, Hamburg, Germany) at 8819 rpm for 10 min at 4 °C. Finally, EE supernatant was filtered with 0.22 mm syringe filters and stored at 4 °C until chemical characterization and use.

### 2.5. Chemical Composition of Essential Oils and Ethanolic Extracts

The chemical composition of the EOs was analyzed in triplicate by gas-liquid chromatography following the procedure described by Herraiz-Peñalver et al. [34]. GC-FID analyses were carried out using a Varian 430-GC gas chromatograph operated with a split/splitless injector (Varian Inc., Palo Alto, CA, USA). Column VF-5ms (polydimethylsiloxane): 60 m length, 0.25 mm internal diameter and 0.25 μm film thickness (Varian Inc., Palo Alto, CA, USA) was used. Temperature program was from 70–95 °C at 3 °C/min, from 96–240 °C (5 min) at 4 °C/min and end temperature was 240 °C for 1 min. Injection and detector (flame ionization, FID) temperatures were 250 and 300 °C, respectively. Injection volume was 0.5 μL with carrier gas helium at 1 mL/min. Injection mode: split at 1:100. Quantification of components (relative percentage abundance) was determined according to the area of the chromatographic peaks using Galaxie^®^ Chromatography software (Varian, Inc. 2002–2005). 

Low molecular weight phenolic compounds (LMWPC) of the EEs were determined according to Cebrián-Tarancón et al. [35]. HPLC grade solvents used were water/formic acid/acetonitrile (97.5:1.5:1 *v*/*v*/*v*) as solvent A and acetonitrile/formic acid/solvent A (78.5:1.5:20 *v*/*v*/*v*) as solvent B. The elution gradient was set up for solvent B as: 0 min, 5%; 8.4 min, 5%; 12.50 min, 10%; 19 min, 15%; 29 min, 16%; 30 min, 16.5%; 34.8 min, 18%; 37.2 min, 32%; 42 min, 62%; 52 min, 90%; 54 min, 100%; 56 min, 100%; 60 min, 5%; 65 min, 5%. Twenty microliters of ethanoic extracts were injected into an Agilent 1200 HPLC chromatograph (Palo Alto, CA, USA) equipped with a Diode Array Detector (Agilent G1315D), coupled to an Agilent ChemStation (version B.03.01) data-processing station. The HPLC column used was ACE 3 C18-PFP with dimensions of 150 × 4.6 mm id (Advance Chromatography Technologies Ltd., Aberdeen, Scotland), and the quantification was based on calibration curves of the respective standards at five different concentrations achieved by UV–vis signal (*R*^2^ = 0.92–0.99). All analyzes were made in duplicate.

### 2.6. Screening of Antifungal Activity from Aromatic Plant Extracts

The antifungal activity of EOs and EEs was evaluated in duplicate with the agar-well diffusion method described by Tepe et al. [36] with slight modifications. EOs were weighed and dissolved in 25% *v*/*v* Tween 20 (Guinama S.L.U., Valencia, Spain) to reach final concentrations of 0.1, 0.5, 1.0, 2.5, 5.0 and 10.0 mg/mL and EEs were assayed without dilution. Each mold strain was cultured for 7 d at 25 ± 1 °C and collected with Tween 20, suspended in sterile saline solution (0.85% *w*/*v*) and diluted at 1–2 × 10^6^ colony forming units (CFU)/mL. Then, 100 µL of each inoculum were spread onto the surface of PDA plates and wells of 10 mm diameter were cut from the agar. Next, 100 µL of the extracts (diluted EOs and pure EEs) were added into the wells. All the plates were incubated for 3 days at 25 ± 1 °C and were examined for halos of growth inhibition. Finally, the images of the plates were obtained with the Countermat flash instrument (IUL S.A., Barcelona, Spain) and the diameter of inhibition zones was measured four times in millimeters using ImageJ v1.52a software. Amphotericin B (Sigma-Aldrich, St. Louis, MO, USA) was used as a positive inhibition control (10 µg/mL), Tween 20 and ethanol 60% *v*/*v* were used as a negative control for EOs and EEs, respectively, and the growth inhibition as a percentage was calculated with the following Equation (1):

% Growth inhibition = [(A_c_ − A_e_)/A_c_] × 100 (1)

where A_c_ is the growth zone of the negative control and A_e_ is the growth zone of the extracts. All the analyses were performed in duplicate.

### 2.7. Determination of Minimum Inhibitory Concentration (MIC) and Minimum Fungicidal Concentration (MFC)

Microdilution broth method according to the Clinical and Laboratory Standards Institute (CLSI) reference protocol M38-A2 for filamentous fungi with modifications [37] was used to evaluate the Minimal Inhibitory Concentrations (MICs) of the extracts that showed antifungal activity. Briefly, dilutions of test essential oils at various concentrations ranging from 0.0098 to 5 mg/mL were prepared with RPMI-1640 medium (with L-glutamine, without bicarbonate) and buffered to pH 7 with 0.164 M MOPS buffer (Sigma-Aldrich, St. Louis, MO, USA) and DMSO (Panreac, Barcelona, Spain) which final concentrations never exceeding 5% *v*/*v*. A 100 microliters solution of each essential oil concentration and 100 µL of tested molds suspension (1–2 × 10^6^ CFU/mL) were added to each well of the 96-well microtiter plates and then incubated at 25 °C for 72 h. Wells containing mold inoculum and RPMI 1640 instead of essential oils served as growth control. MIC was defined as the lowest concentration of the essential oils at which no visible growth of the fungal strain could be detected compared to their growth in the negative control well. Tests were performed in duplicate and visual interpretation was carried out by two qualified individuals. The Minimum Fungicidal Concentrations (MFCs) were determined according to Espinel-Ingroff et al. [38] by subculturing 10 µl of the wells with no mold growth onto plates containing PDA agar and incubating at 25 °C for 72 h. The lowest concentration of EOs with negative growth was considered to be the MFC. The isolates were tested in duplicate.

### 2.8. Antioxidant Capacity of Extracts from Aromatic Plants 

#### 2.8.1. Total Phenolic Content (TPC) 

The Total Phenolic Content (TPC) of the aromatic plant extracts was determined in triplicate using Folin-Ciocalteu reagent as described by Singleton and Rossi [39] with modifications. Briefly, 200 µL of EOs and EEs dissolved in methanol (1 mg/mL) were mixed with 2.5 mL of Folin-Ciocalteu (Sigma-Aldrich, St. Louis, MO, USA) diluted 1:10 in distilled water. The samples were incubated at room temperature for 5 min and vortexed. Next, two mL of 7.5% *w*/*v* Na_2_CO_3_ (Sigma-Aldrich, St. Louis, MO, USA) was added and the tubes were incubated for 90 min in darkness. The absorbance of samples was measured at 765 nm using a spectrophotometer UV-Vis GENESYS 150 (Thermo Fisher Scientific, Waltham, MA, USA) against a blank of distilled water. A standard curve with concentrations of gallic acid in methanol ranging from 0.05 to 2 mg/mL was performed. The amount of TPC was determined as milligrams of gallic acid equivalent per gram of extract (mg GAE/g extract) using the standard curve.

#### 2.8.2. Scavenging Activity of DPPH Radicals

The 1,1-diphenyl-2-picrylhydrazyl (DPPH) radical scavenging activity of plant extracts was determined following the method described by Braca et al. [40] with minor modifications. An aliquot of 0.6 mL of the EOs (0.05–50 mg/mL) and EEs (0.5–20 mg/mL) was mixed with 2.4 mL of DPPH in methanol (0.004% *w*/*v*), shaken and incubated for 30 min in darkness. Then, the absorbance was measured at 517 nm by a spectrophotometer. Acid gallic (Sigma-Aldrich, St. Louis, MO, USA) was used as standard antioxidant. The inhibition activity (%) of DPPH radicals was calculated as follows (2):

DPPH inhibition (%) = [(A_0_ − A_1_)/A_0_] × 100 (2)

where A_0_ was the absorbance of DPPH, and A_1_ was the absorbance of the samples. The concentration of sample required to reduce 50% of DPPH radicals (IC_50_) was calculated from linear regression (% inhibition DPPH vs. concentration of the samples, *R*^2^ ≥ 0.90). The samples were measured in duplicate.

### 2.9. Statistical Analysis

Statistical analysis of data was performed using SPSS (IBM SPSS Statistics version 26). ANOVA (one way), calculated using a confidence level of 95% to determine any significant difference between the aromatic plant extracts. When there was a significant difference (*p* < 0.05) the Tukey test was carried out to determine differences between the antifungal activity of the EOs.

## 3. Results

### 3.1. Identification of Molds Isolated from Sheep Cheese

Among the gene markers used in *Penicillium* identification, the β-tubulin gene has been recommended as a specific barcode for species identification [16,41]. In the present work, a total of 14 isolates of the 21 collected were successfully cultivable under laboratory conditions and identified at species level by DNA barcoding (β-tubulin sequence). As Table 1 shows, the most common isolates were *Penicillium* (12 isolates, 85.7%) and *Aspergillus* (2 isolates, 14.3%); both belong to Ascomycota phylum (Pezizomycotina subphylum, class Eurotiomycetes) and are considered as the main food spoilage molds [5]. Several studies reported *Penicillum* and *Aspergillus* as the predominant genus found in cheese and dairy industries [7,8,42,43,44,45,46,47]. Moreover, the use of β-tubulin amplification and sequencing allowed the separation of closely related species of *Penicillium* and *Aspergillus*. As presented in Table 1, the isolates identified were: *P*. *crustosum* (seven isolates, 50%), *P*. *commune*/*biforme* (five isolates, 35.7%), *A*. *puulaauensis* (one isolate, 6.3%) and *A*. *jensenii* (one isolate, 6.3%). Based on the classification proposed by Houbraken and Samson [48] and Visagie et al. [16], all *Penicillium* isolates belonged to the *Fasciculata* section and within them, *P*. *commune*, *P*. *biforme*, and *P*. *crustosum* were the most frequent. The high presence of *P*. *commune* in cheese is due to its ability to grow at low temperatures, its lipolytic activity, its low oxygen concentrations and its resistance to the action of some preservatives [49] even though none of the cheeses used for the sampling had been treated with any type of preservative. Other studies have found *P*. *commune* as one of the most common spoilage molds in sheep and goat cheeses [43], Manchego PDO [50], Spanish semi-hard ripened cheese [11], kuflu cheese [44], Italian grana cheese [47] and semi-hard cheese [51]. In the past, *P*. *biforme* was considered as synonymous with *P*. *camemberti* and *P*. *commune* [28], but it was redefined as distinct species by Giraud et al. [41]. 

Given this information and considering the phenotypic and ecological characteristics [11], our strains could not be *P*. *camemberti*, since it usually grows as a white and floccose mycelium and the mycelia observed for all isolates were green-grey. In comparison with other studies, it was observed that *P*. *biforme* was also isolated from traditional artisan Italian cave cheese by Anelli et al. [52]. Moreover, a recent study carried out by Ropars et al. [53] suggests that wild strains identified in the literature as *P*. *commune* correspond to either *P*. *fuscoglaucum* or *P*. *biforme*, differentiated according to the isolation environment (*P*. *fuscoglaucum* is isolated from natural environments and *P*. *biforme* is commonly isolated from cheese). (Due to the changing situation in the taxonomy of these species, we will use the term *P*. *commune*/*biforme* to refer our isolates from now on).

Seven isolates were identified as *P*. *crustosum* (Table 1), which is a mold that can cause spoilage in several foods such as cheese, meat and refrigerated foods [54] and has been commonly isolated and identified in cheeses by other authors [28,43,45,46,47,51,55]. 

Finally, two isolates were identified as *A*. *puulaauensis* and *A*. *jensenii*, species belonging to the *Versicolores* section [56], and to the best of our knowledge this is the first time that these molds have been isolated from semi-hard sheep cheeses. However, there are other studies in which these molds were isolated from cheese such as Italian grana [46], Italian cave [52] and gouda and cheddar [45] made from cow’s milk. 

**Figure 1:**
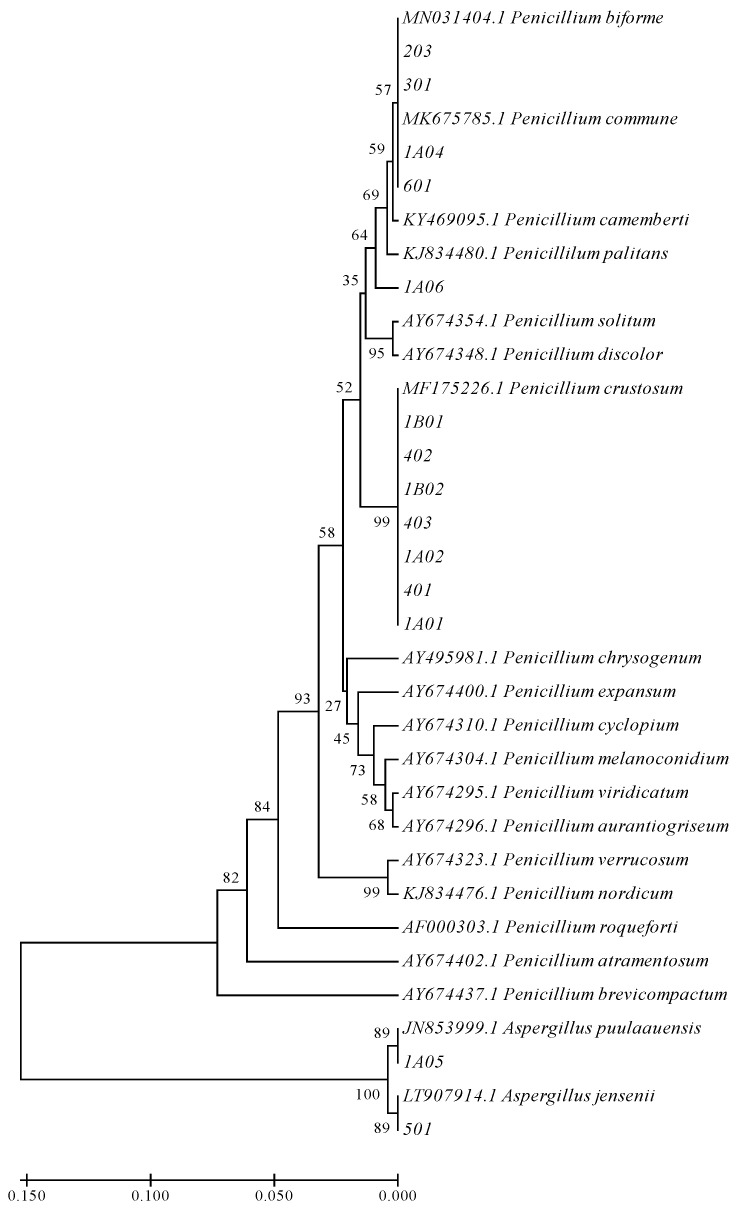
Phylogenetic tree obtained after UPGMA analysis of the β-tubulin BenA sequences. The accession number of the verified sequences used for comparison purposes is indicated. The percentage value of “Bootstrap” is indicated in the nodes of the branches.

Furthermore, BenA phylogenetic analysis (Figure 1) shows the relationship between the isolated strains (coded with numbers) and the verified strains obtained from the NCBI database for β-tubulin sequences commonly found in cheeses. The results confirmed the molecular identification of the strains and showed that *P*. *commune*, *P*. *biforme* and *P*. *camemberti* belong to the same cluster. Likewise, the *BenA* phylogenetic tree shows that both *P*. *commune* and *P*. *crustosum* belong to the series *Camemberti* and are closely related taxons (Figure 1). 

The fact that the molds were isolated from different farms, as well as the type of milk used in the production of cheese, such as isolate 203 that comes from cheese made from heat-treated milk, might indicate that those isolates identified as the same species are in fact different strains.

### 3.2. Mycotoxin Production

The result of the mycotoxin analysis (OTA, PAT, CPA and STC) of the strains showed that all *P*. *commune*/*biforme* isolates were CPA producers. Besides, *A*. *jensenni* and *A*. *puulaauensis* produced STC but the two mycotoxins were detected at levels below 10 and 50 μg/mL, respectively (data not shown). Other studies established that *P*. *commune* and its domesticated species *P*. *camemberti* are the main producers of CPA in cheese [7,11,14]. Cyclopiazonic acid is a mycotoxin that causes necrosis in vertebrate inner organs at high concentrations [14], but it does not possess potent acute toxicity since its oral LD_50_ for rodents is 30–70 mg/kg. Although there is no regulation on CPA levels in foods, Finoli et al. [57] reported that the concentrations of CPA that can be found in cheeses would not be dangerous for the consumer. Moreover, Burdock and Flamm [58] stated in their review of cyclopiazonic safety that, considering the limit of CPA found in cheeses is 4 µg/g and average consumption is 50 g of cheese per day, this cheese intake would not exceed the established NOEL (1 mg/kg/day).

Other authors reported that *A*. *jensenii* and *A*. *puulaauensis* are producers of STC [29] and this mycotoxin has been previously found in cheeses, but produced by *A*. *versicolor* [59]. STC is a polyketide mycotoxin that can cause acute toxicity in liver and kidneys [60]; however, the risk of STC to human health has not been characterized as exposure data are limited. In 2013, the Panel on Contaminants in the Food Chain (CONTAM) from EFSA published a report on the risk of STC for public health and concluded that exposure to this mycotoxin is a low concern based on its relative carcinogenic potency and exposure data [60]. Finally, ochratoxin A (OTA) and patulin (PAT) production was not detected. However, the presence of other types of mycotoxins in sheep cheeses such as aflatoxin M1 has been previously reported by other authors, but below the maximum levels allowed by the European Union in milk (50 ng/kg) [61].

### 3.3. Chemical Composition of Aromatic Plant Extracts

The results from the analysis of the five-steam distilled EOs by GC–FID, represented as retention time and percentage composition, as well as the presence of 71 compounds identified, are given in Table 2. The analysis of the constituents of *Artemisia dracunculus* EO revealed that the major components were methyl eugenol (38.72%), elimicin (26.12%), (z)-iso-elemicin (20.24%), cis-methyl-isoeugenol (2.87%), terpinen-4-ol (2.08%) and spathulenol (1.44%). The EO of *Hyssopus officinalis* was mainly composed of 1,8-cineole (48.23%), ß-pinene + mircene (16.36%), Limonene (6.02%), isopino-camphone (4.38%), pino-camphone (3.42%), 1-octen-3-ol (3.32%), pino-carvone (3.16%) α-pinene (2.67%), ß-E-ocimene (1.97%), α-terpineol (1.87%), valencene (1.25%) and p-cymene (1.25%). As shown in Table 2, the major compounds found in *Lavandula stoechas* EO were linalool (33.79%), 1,8-cineole (20.59%), α-pinene (12.11%), camphor (9.56%), limonene (3.73%) and ß-pinene + myrcene (1.15%). Finally, the analysis of *Origanum vulgare* EO revealed that its main components were carvacrol (82.42%), thymol (4.98%), trans-caryophyllene (2.70%), p-cymene (2.43%) and γ-terpinene (1.82%), and those of *Satureja montana* EO were carvacrol (77.59%), p-cymene (6.62%), iso-borneol (2.23%), γ-terpinene (1.28%), linalool (1.23%) and α-trans-bergamotene (1.01%).

The chemical composition of essential oils can vary greatly depending on the geographical origin of the plant, the time of harvest and the part of the plant used in extraction [62].Comparing with other works, it was observed that the major components of *H*. *officinalis* EO (1,8-cineole and ß-pinene + myrcene) were comparable to the literature [63,64], but the percentages of carvacrol obtained in the EOs of *S*. *montana* (77.59%) and *O*. *vulgare* (82.41%) were higher than those found by other authors [65,66]. The major compounds present in *A*. *dracunculus* EO have been previously reported by other authors in similar percentages [67,68] but the amount of linalool (33.79%) present in *Lavandula stoechas* differs from that in other works [69].

For the ethanolic extracts, LMWPC of the EEs obtained from solid by-products of the aromatic plant distillation were protocatechuic acid, syringic acid, caffeic acid, and sinapaldehyde (Table 3). It was noticed that caffeic acid was one of the major LMWPCs in all the samples with the highest amount obtained in the EE from *A*. *dracunculus* (29.07 mg/L). Moreover, the highest concentration of protocatechuic acid was found in the EE of *O*. *vulgare* (52.26 mg/L) and sinapaldehyde was only present in the EEs obtained from the solid-by-product of *S*. *montana* and *O*. *vulgare* with concentrations of 12.40 and 15.68 mg/L, respectively.

Caffeic acid and protocatechuic acid have been previously found in extracts of solid distillation residues of *R*. *officinalis* and Lavandin by Santana-Méridas et al. [27] and Torras-Claveria et al. [70], respectively. However, research into the phenolic composition of ethanolic extracts obtained from steam distilled solid by-products from aromatic plants is still limited and should be further investigated, since several phenolic compounds such as those identified in the present work are widely known for having antioxidant activity [71,72,73,74] and could be valuable for the agri-food industry.

### 3.4. Antifungal Activity of Aromatic Plant Extracts

As presented in Table 4, the results showed that *O*. *vulgare*, *S*. *montana* and *A*. *dracunculus* EOs had remarkable antifungal activity against all the isolated strains at the highest assayed concentration (10 mg/mL) providing large growth inhibition halos (25–51 mm). By contrast, any of the EEs or EOs from *H*. *officinalis* or *L*. *stoechas* showed antifungal activity at these concentrations, which is why they are not included in Table 4. The EOs from *O*. *vulgare* and *S*. *montana* showed significantly higher inhibition halos (28–45 mm and 32–51 mm, respectively) than those obtained for *A*. *dracunculus* (25–40 mm). Oregano and savory EOs showed very similar percentages of inhibition except for *A*. *puulaauensis* (1A05) and *P*. *crustosum* (1B01) which were significantly (*p* < 0.001) higher in *S*. *montana*. On the other hand, *P*. *crustosum* (401, 403 and 1B02), and *P*. *commune* (1A06) were significantly higher (*p* < 0.001) when using *O*. *vulgare*.

Likewise, when comparing the three EOs, Table 4 shows that *A*. *dracunculus* is the weakest in the majority of the strains, except *P*. *crustosum* (1A02) where there were no significant differences (*p* > 0.05) among the three EOs.

Considering the strains at species level, it was observed that the most sensitive to the action of *O*. *vulgare* and *S*. *montana* EOs were all *P*. *commune*/*biforme* strains, *A*. *puulaauensis*, *A*. *jensenii* and *P*. *crustosum* (1B02) with percentages of growth inhibition between 41% and 57%. By contrast, the most resistant to these essential oils were the rest of the *P*. *crustosum* strains (1A01, 1A02, 1B01 and 402) with percentages of inhibition ranging from 31% to 38%. Besides, the maximum antifungal capacity was reached using *O*. *vulgare* EO on *P*. *crustosum* (401) with a 50% inhibition, and on *A*. *puulaauensis* with *S*. *montana* EO reaching 57% inhibition of mold growth.

The origin of the mold strains and the fact of being isolated from cheese made with pasteurized or raw milk (Table 1) appeared to have had some influence on the effectivity of the essential oils since the inhibition percentages vary between molds of the same species (Table 4), thus confirming that they could be different strains. Furthermore, it was observed that oregano and savory essential oils also caused changes in the pigmentation and sporulation of the tested molds and this fact might be because the mode of action of the essential oil against the fungi includes an attack on their cell wall which can result in the death of the mycelium [75]. The powerful antifungal activity of oregano and savory EOs could be explained due to the high amount of carvacrol that they contain (Table 2). It is well stablished that carvacrol is a phenolic compound with powerful antimicrobial properties [76] and other authors have related the strong antifungal activity of oregano and savory essential oils with the large amounts of carvacrol and thymol in their composition [65]. Regarding tarragon EO, it has been previously reported that its main components (methyl eugenol and elimicin) possess antimicrobial activity against a wide range of molds and other microorganisms [77,78].

Ethanolic extracts are mainly composed of low molecular weight phenolic compounds (Table 3) and, even though traces of several compounds present in the corresponding essential oils have been found in these extracts (data not shown), the compounds with antifungal activity remain mostly in the EOs. Therefore, it is suspected that the low concentration of these compounds is not sufficient to provide the extract with antifungal activity.

The results obtained from MIC and MFC determination of the three active EOs are shown in Table 5. As observed in the agar-well diffusion test, when MIC was calculated by microdilution assay it was confirmed that the essential oil of *S*. *montana* was the most effective, followed by *O*. *vulgare* and *A*. *dracunculus*.

High fungistatic and fungicidal activity of savory and oregano EOs was demonstrated with low MIC and MFC values. The mean MIC value was slightly higher in savory (0.52 mg/mL) than in oregano EO (0.43 mg/mL), the highest obtained being that of tarragon essential oil (0.85 mg/mL).

It was noticed that mean MIC and MFC values were the same for *S*. *montana* (0.52 mg/mL) and that the latter was slightly lower than oregano MFC (0.58 mg/mL).

The highest MIC and MFC mean values (0.85 and 1.12 mg/mL, respectively) were obtained for *A*. *dracunculus* EO. This is in concordance with the results obtained in the agar-well diffusion assay (Table 4) and demonstrate that tarragon EO has lower fungicidal and fungistatic capacity than *O*. *vulgare* and *S*. *montana* EOs.

Regarding the strains at species level, *P*. *commune* (203) was the most susceptible strain to all the essential oils, and fungicidal effect was achieved in ranges from 0.08 to 0.31 mg/mL. On the other hand, the most resistant strains were those identified as *P*. *commune*/*biforme* (1A04, 601) requiring a concentration of 2.5 mg/mL to achieve the fungicidal effect (Table 5).

Other authors observed a strong antifungal capacity of savory EO against *A*. *flavus*, *A*. *niger*, *Fusarium* species and *Penicillium* sp. with low MIC values ranging from 0.07 to 0.95 mg/mL [65]. Moreover, Camiletti et al. [79] observed low MFC (0.6–1.1 mg/mL) and MIC (0.4–0.5 mg/mL) values in oregano EO against *A*. *flavus*, *P*. *oxalicum* and *P*. *minioluteum*, and Behbahani et al. [80] reported similar MIC values for tarragon EO against *A*. *fumigatus* (2 mg/mL) and *P*. *expansum* (4 mg/mL).

### 3.5. Antioxidant Capacity of Essential Oils and Ethanolic Extracts from Aromatic Plants

The antioxidant profile of the aromatic plant extracts tested in this work is presented in Table 6.

The essential oils with the highest TPC were oregano (100.87 mg GAE/g extract), savory (73.31 mg GAE/g DW) and tarragon (23.77 mg GAE/g extract) followed by hyssop and Spanish lavender, which had similar values (10.67 and 9.31 mg GAE/g extract, respectively). The differences found in the TPC of oregano and savory essential oils (whose major compound is carvacrol) may be due to the fact that oregano EO contains almost 5% more than savory EO, and to the presence of 5% thymol in oregano essential oil (Table 2).

An overall increase in TPC content was observed in all aromatic plants for the EEs compared to the Eos, although the behavior was slightly different, with oregano and Spanish lavender standing out, followed by tarragon, savory and hyssop. Besides, it was observed that TPCs were much lower in the EOs than in the EEs and this may be related to the LMWPC present in the ethanolic extracts (Table 3). On the other hand, the extract with the highest TPC content was the EE from *O*. *vulgare* (341.19 mg GAE/g extract) and the lowest the EO from *L*. *stoechas* (9.31 mg GAE/g extract). 

The extracts with the highest antioxidant capacity measured as reduction of the DPPH radical were oregano and savory EOs with an IC_50_ of 0.07 and 0.09 mg/mL, respectively, followed by tarragon EO with an IC_50_ of 1.18 mg/mL. Hyssop and Spanish lavender EOs had the highest IC_50_ values (29.91 and 14.80 mg/mL, respectively). A similar behavior was observed in the EEs, with the lowest IC_50_ values obtained in oregano (0.12 mg/mL), savory (2.32 mg/mL) and Spanish lavender (2.97 mg/mL) and the highest with tarragon (4.86 mg/mL) and hyssop (9.24 mg/mL). The low IC_50_ values of oregano, savory and tarragon essential oils are mainly due to the presence of carvacrol and methyl eugenol in their composition (Table 2) since other authors have related these compounds to low IC_50_ values [81,82]. The lowest IC_50_ value of the ethanolic extracts was obtained with oregano EE and this may be due to the fact that this contains the highest amount of LMWPC (Table 3).

Among all the evaluated extracts, *O*. *vulgare* EO and its EE have the highest antioxidant capacity, even equal to that of the standard antioxidant gallic acid (0.06 mg/mL). Proestos et al. [83] have previously reported the strong antioxidant capacity of the oregano EO with values comparable to those obtained in the present study (0.08 mg/mL vs. 0.07 mg/mL).

The results for TPC content and scavenging activity of DPPH radical’s analysis proves that EEs contain higher amounts of TPC than EOs and, although in some cases the IC_50_ of the ethanolic extracts were slightly higher, they still demonstrated a remarkable antioxidant capacity. These data support that ethanolic extracts are a valuable source of bioactive compounds which may be useful in the development of new functional foods and would also help to promote the circular economy in the agri-food and essential oil industry by utilizing a by-product from the distillation of essential oils.

## 4. Conclusions

The molecular identification of the mold strains isolated from sheep cheese reported the presence of two species of *Aspergillus* (*A*. *jensenii* and *A*. *puulaauensis)* never found in sheep cheeses, that showed the ability to produce sterigmatocystin, but at a level inferior to 50 μg/mL. Another two species, *P*. *commune*/*biforme* and *P*. *crustosum*, were found growing on the surface of this type of cheese during ripening, highlighting the capacity of all the *P*. *commune*/*biforme* strains to produce cyclopiazonic acid (<10 μg/mL). The control of spoilage molds plays a key role in the dairy industry, so the findings of the present study will contribute to the identification and characterization of the sheep cheeses’ fungi, allowing more effective control measures, in order to secure the safety of consumers and reduce economic losses.

Moreover, this work confirms that *Satureja montana*, *Origanum vulgare* and *Artemisia dracunculus* EOs have high antifungal activity, the first two being more powerful against all the tested strains with lower MIC and MFC values. Their EOs and EEs were the extracts with the lowest IC_50_ values, and the total phenolic content was superior in the EEs compared to the amounts present in EOs.

This work demonstrates that *Origanum vulgare*, *Satureja montana* and *Artemisia dracunculus* EOs have great potential in fungal growth control and might be a suitable alternative to chemical antimicrobials due to their natural origin and eco-friendly aspects, in addition to a broad spectrum of action, and are considered safe for human consumption.

Despite the advantages of essential oils over the use of chemical preservatives and their proven potential application as fungal inhibitors, one of the main challenges faced by the food industry is their impact on the organoleptic properties of foods when applied directly. Such issues can be overcome by the incorporation of EOs into edible films/coatings or their nanoencapsulation in small-size particles to meet dairy industry needs.

Likewise, the results of this research might indicate that the use of aromatic plants can be extended after their EO distillation, by obtaining ethanolic extracts with strong antioxidant capacities that could be advantageous as an option for use in the development of functional foods rich in antioxidant compounds.

## Figures and Tables

**Table 1:** Origin and identification of molds isolated from sheep cheese by amplification of β-tubulin BenA gene.

Mold Code	Farm Code	Type of Cheese Milk	Fungal Strain
1A01	1A	Raw	*Penicillium crustosum*
1A02	1A	Raw	*Penicillium crustosum*
1A04	1A	Raw	*Penicillium commune*/*biforme*
1A05	1A	Raw	*Aspergillus puulaauensis*
1A06	1A	Raw	*Penicillium commune*/*biforme*
1B01	1B	Raw	*Penicillium crustosum*
1B02	1B	Raw	*Penicillium crustosum*
203	2	Pasteurized	*Penicillium commune*/*biforme*
301	3	Raw	*Penicillium commune*/*biforme*
401	4	Raw	*Penicillium crustosum*
402	4	Raw	*Penicillium crustosum*
403	4	Raw	*Penicillium crustosum*
501	5	Raw	*Aspergillus jensenii*
601	6	Raw	*Penicillium commune*/*biforme*

**Table 2:** Chemical composition of essentials oil from aromatic plants (area %).

Compound	RT^1^ (min)	*Artemisia* *dracunculus* (Tarragon) (%)	*Hyssopus* *officinalis* (Hyssop) (%)	*Lavandula* *stoechas* (Spanish Lavender) (%)	*Origanum* *vulgare* (Oregano) (%)	*Satureja* *montana* (Savory) (%)
tricyclene	11.00	-	0.19	0.07	0.41	-
α -thujene	11.06	-	-	0.11	-	-
α-pinene	11.36	-	2.67	12.11	0.21	0.08
camphene	11.98	-	0.08	0.50	-	0.07
1-octen-3-ol	12.63	0.25	3.32	0.97	0.24	0.87
sabinene	12.82	-	-	0.08	0.09	-
ß-pinene + mircene	12.95	-	16.36	1.15	0.69	0.26
delta-3-carene	13.71	-	0.078	0.09	0.06	-
3-carene	13.91	-	-	4.20	-	-
α -terpinene	14.15	-	0.08	0.33	0.40	0.38
p-cymene	14.44	-	1.25	0.81	2.43	6.62
limonene	14.65	-	6.02	3.73	0.09	0.06
1,8-cineole	14.93	-	48.23	20.59	-	0.28
ß-e-ocimene	15.00	-	1.97	0.06	-	-
γ-terpinene	15.61	-	0.27	0.18	1.82	1.28
cis-sabinene hydrate	16.15	-	0.24	0.29	0.09	0.26
terpinolene	16.66	-	0.13	0.18	0.06	0.06
linalool	16.98	0.25	0.17	33.79	0.11	1.23
α-thujone	17.53	-	-	0.05	-	-
ß-thujone	18.01	-	0.07	0.30	-	0.05
camphor	19.31	-	0.75	9.56	-	-
pinocamphone	19.70	-	3.42	-	-	-
pinocarvone	19.77	-	3.16	-	-	-
ß-pinene oxide	19.90	-	0.28	0.47	-	-
isoborneol	20.11	-	-	-	-	2.23
borneol	20.21	0.17	0.18	0.61	0.26	0.12
isopinocamphone	20.31	-	4.38	-	-	-
terpinen-4-ol	20.31	2.08	-	0.42	0.70	0.95
α-terpineol	20.80	0.37	1.87	0.37	0.08	0.14
myrtenol	20.96	0.15	0.20	0.16	0.08	-
verbonene	21.34	-	-	0.35	0.06	-
trans-carveol	21.61	0.45	-	0.12	-	-
nerol	22.11	-	-	-	-	0.05
linalyl acetate	22.32	0.13	-	0.21	-	-
geraniol	22.58	0.31	-	0.12	-	-
geranial	23.08	-	-	0.08	-	-
bornyl acetate	23.94	0.72	-	0.17	-	0.44
thymol	24.15	-	0.05	-	4.98	-
carvacrol	24.55	-	-	-	82.42	77.59
terpin-4-ol acetate	24.98	-	-	0.09	-	-
α-terpinyl acetate	25.91	-	0.14	-	-	-
α-cubebene	26.20	0.27	-	-	-	0.053
geranyl acetate	26.68	0.55	-	-	-	-
α-copaene	27.09	-	-	0.09	-	0.08
ß-elemene	27.42	-	0.38	-	-	0.06
ß-cubebene	27.56	-	0.08	-	-	0.46
methyl eugenol	27.74	38.72	-	-	-	-
α-gurjunene	28.19	-	0.08	-	-	-
α-trans-bergamotene	28.65	-	0.32	-	-	1.01
trans-caryophyllene	28.68	-	-	-	2.70	-
z-ß-farnesene	29.06	-	-	-	0.14	0.14
aromadendrene	29.25	-	0.21	-	-	0.11
e-ß-farnesene	29.80	-	0.07	0.05	0.22	0.06
ar-curcumene	30.23	-	-	-	-	0.20
valencene	30.56	-	1.25	0.22	-	0.17
cis-methyl-isoeugenol	30.66	2.87	-	-	-	-
γ-muurolene	31.01	-	0.22	-	0.49	0.53
cubebol	31.52	-	-	-	-	0.27
delta-cadinene	31.69	-	-	-	-	0.07
elimicin	32.25	26.12	-	-	-	-
ß-sesquiphellandrene	32.46	0.29	0.20	-	-	0.55
germacrene d	33.54	-	0.10	-	-	0.27
spathulenol	33.58	1.44	-	-	-	-
cariophyllene oxide	33.83	0.29	0.06	-	0.57	0.52
viridiflorol	34.11	-	0.07	-	-	-
humulene epóxido ii	35.02	-	-	-	-	0.05
(z)-isoelemicin	35.19	20.24	-	-	-	-
cubenol	35.28	-	-	-	-	0.06
ß-eudesmol	35.93	0.14	-	-	-	-
α-eudesmol	36.13	-	-	-	-	0.08
geranyl tiglate	36.81	0.12	-	-	-	-
Total	96.31	98.86	93.47	99.40	97.77	

RT^1^: Retention time.

**Table 3:** Low molecular weight phenolic compounds (LMWPC) of ethanolic extracts from aromatic plant by-products (mg/L) (mean ± sd).

Compound	*Artemisia* *dracunculus* (Tarragon) (mg/L)	*Hyssopus officinalis* (Hyssop) (mg/L)	*Lavandula* *stoechas* (Spanish Lavender) (mg/L)	*Origanum vulgare* (Oregano) (mg/L)	*Satureja* *montana* (Savory) (mg/L)
Protocatechuic acid	2.07 ± 0.04	1.36 ± 0.01	3.48 ± 0.09	52.26 ± 0.20	2.80 ± 0.03
Syringic acid	-	3.04 ± 0.11	1.76 ± 0.01	-	6.74 ± 0.18
Caffeic Acid	29.07 ± 0.34	14.23 ± 0.71	14.76 ± 0.05	13.68 ± 0.14	22.84 ± 0.20
Sinapaldehyde	-	-	-	15.68 ± 0.36	12.40 ± 0.66

**Table 4:** Inhibition halos (mm) and percentage of inhibition (%) of essential oils against the isolated mold strains (mean ± sd).

Code	Strain	WD (mm) ^1^			% Inhibition		
		*Artemisia* *dracunculus* (Tarragon)	*Origanum* *vulgare* (Oregano)	*Satureja* *montana* (Savory)	*Artemisia* *dracunculus* (Tarragon)	*Origanum* *vulgare* (Oregano)	*Satureja* *montana* (Savory)
1A01	*P*. *crustosum*	26.45 ± 0.55 ^a^	30.21 ± 2.81 ^ab^	33.68 ± 1.62 ^b^	29.39 ± 0.61 ^a^	33.56 ± 3.12 ^ab^	37.42 ± 1.79 ^b^
1A02	*P*. *crustosum*	31.05 ± 0.51	30.24 ± 1.69	31.86 ± 0.70	34.50 ± 0.56	33.60 ± 1.88	35.39 ± 0.78
1A04	*P*. *commune* *	30.17 ± 2.80 ^a^	38.63 ± 0.47 ^b^	41.89 ± 0.94 ^c^	33.53 ± 3.11 ^a^	42.93 ± 0.53 ^b^	46.54 ± 1.04 ^c^
1A05	*A*. *puulaauensis*	32.56 ± 0.75 ^a^	43.16 ± 1.19 ^b^	51.32 ± 1.80 ^c^	36.18 ± 0.84 ^a^	47.96 ± 1.32 ^b^	57.02 ± 2.00 ^c^
1A06	*P*. *commune* *	26.27 ± 0.41 ^a^	37.88 ± 0.71 ^c^	35.36 ± 0.53 ^b^	29.19 ± 0.45 ^a^	42.09 ± 0.79 ^c^	39.28 ± 0.59 ^b^
1B01	*P*. *crustosum*	25.24 ± 1.59 ^a^	28.40 ± 2.07 ^b^	32.93 ± 0.91 ^c^	28.05 ± 1.77 ^a^	31.55 ± 2.30 ^b^	36.58 ± 1.01 ^c^
1B02	*P*. *crustosum*	27.78 ± 1.10 ^a^	42.87 ± 1.95 ^c^	36.85 ± 0.69 ^b^	30.87 ± 1.23 ^a^	47.63 ± 2.16 ^c^	40.95 ± 0.77 ^b^
203	*P*. *commune* *	28.77 ± 1.93 ^a^	37.26 ± 1.81 ^b^	39.07 ± 1.56 ^b^	31.97 ± 2.15 ^a^	41.40 ± 2.02 ^b^	43.41 ± 1.73 ^b^
301	*P*. *commune* *	31.67 ± 1.46 ^a^	40.75 ± 0.31 ^b^	38.78 ± 0.45 ^b^	35.19 ± 1.62 ^a^	45.28 ± 0.34 ^b^	43.08 ± 0.50 ^b^
401	*P*. *crustosum*	39.93 ± 0.72 ^a^	45.47 ± 1.06 ^b^	41.66 ± 1.36 ^a^	44.37 ± 0.80 ^a^	50.52 ± 1.17 ^b^	46.29 ± 1.51 ^a^
402	*P*. *crustosum*	27.87 ± 1.13 ^a^	33.26 ± 1.37 ^b^	34.91 ± 1.26 ^b^	30.97 ± 1.26 ^a^	36.95 ± 1.52 ^b^	38.78 ± 1.40 ^b^
403	*P*. *crustosum*	24.25 ± 0.67 ^a^	38.63 ± 0.57 ^c^	33.76 ± 0.71 ^b^	26.94 ± 0.74 ^a^	42.93 ± 0.64 ^c^	37.51 ± 0.79 ^b^
501	*A*. *jensenii*	39.16 ± 0.80 ^a^	42.41 ± 1.25 ^b^	42.08 ± 0.40 ^b^	43.51 ± 0.89 ^a^	47.12 ± 1.39 ^b^	46.76 ± 0.44 ^b^
601	*P*. *commune* *	30.47 ± 0.88 ^a^	40.71 ± 1.16 ^b^	40.71 ± 0.78 ^b^	33.86 ± 0.98 ^a^	45.23 ± 1.29 ^b^	45.23 ± 0.86 ^b^

^1^ WD, agar-well diffusion method. Diameter of inhibition zone (mm) including well diameter of 10 mm; ^a–c^ Different superscripts show significant differences (*p* < 0.05) between essential oils. * *P*. *commune*/*biforme*.

**Table 5:** Minimum inhibitory concentration (MIC) and minimum fungicidal concentration (MFC) of essential oils with antifungal activity expressed in mg/mL.

Code	Strain	*Artemisia* *dracunculus* (Tarragon)		*Origanum* *vulgare* (Oregano)		*Satureja* *montana* (Savory)	
		MIC	MFC	MIC	MFC	MIC	MFC
1A01	*P*. *crustosum*	0.63	0.63	0.63	0.63	1.25	1.25
1A02	*P*. *crustosum*	0.63	0.63	0.63	0.63	0.63	0.63
1A04	*P*. *commune* *	0.63	2.5	0.16	0.63	0.31	0.31
1A05	*A*. *puulaauensis*	0.63	0.63	0.16	0.63	0.31	0.31
1A06	*P*. *commune* *	1.25	1.25	0.63	0.63	0.63	0.63
1B01	*P*. *crustosum*	1.25	1.25	0.63	0.63	0.63	0.63
1B02	*P*. *crustosum*	1.25	1.25	0.31	0.31	0.31	0.31
203	*P*. *commune* *	0.31	0.31	0.08	0.31	0.08	0.08
301	*P*. *commune* *	0.63	0.63	0.63	0.63	0.63	0.63
401	*P*. *crustosum*	1.25	1.25	0.63	0.63	0.31	0.31
402	*P*. *crustosum*	1.25	1.25	0.16	0.63	0.31	0.31
403	*P*. *crustosum*	0.31	0.31	0.63	0.63	0.63	0.63
501	*A*. *jensenii*	1.25	1.25	0.16	0.63	0.63	0.63
601	*P*. *commune* *	0.63	2.5	0.63	0.63	0.63	0.63

* *P*. *commune*/*biforme*.

**Table 6:** Total phenolic content (mg GAE ^1^/g extract) and antioxidant activity of aromatic plant extracts measured by DPPH radical-scavenging assay (IC_50_ ^2^, mg/mL) (mean ± sd).

Aromatic Plant	TPC ^3^		IC^50^	
	EO ^4^	EE ^5^	EO ^4^	EE ^5^
*Artemisia dracunculus*	23.77 ± 1.60	91.19 ± 1.61	1.18 ± 0.18	4.86 ± 0.15
*Hyssopus officinalis*	10.67 ± 0.96	68.94 ± 1.61	29.91 ± 1.75	9.24 ± 0.69
*Lavandula stoechas*	9.31 ± 0.96	143.42 ± 1.61	14.80 ± 1.52	2.97 ± 0.28
*Satureja montana*	74.31 ± 0.34	87.33 ± 1.93	0.09 ± 0.04	2.32 ± 0.18
*Origanum vulgare*	100.87 ± 0.36	341.19 ± 1.28	0.07 ± 0.01	0.12 ± 0.02

^1^ Gallic acid equivalent. ^2^ Concentration sample required to reduce 50% DPPH radicals. ^3^ Total phenolic content. ^4^ Essential oil. ^5^ Ethanolic extract.

## Data Availability

The data presented in this study are available on request from the corresponding author.
